# Molecular Characterization and Antimicrobial Susceptibility of *Staphylococcus aureus* Isolates from Clinical Infection and Asymptomatic Carriers in Southwest Nigeria

**DOI:** 10.1371/journal.pone.0137531

**Published:** 2015-09-08

**Authors:** Olayemi O. Ayepola, Nurudeen A. Olasupo, Louis O. Egwari, Karsten Becker, Frieder Schaumburg

**Affiliations:** 1 Department of Biological Sciences, Covenant University, Ota, Ogun State, Nigeria; 2 Department of Microbiology, Lagos State University, Ojo, Lagos State, Nigeria; 3 Institute for Medical Microbiology, University Hospital Münster, Münster, Germany; Kent State University, UNITED STATES

## Abstract

Few reports from Africa suggest that resistance pattern, virulence factors and genotypes differ between *Staphylococcus aureus* from nasal carriage and clinical infection. We therefore compared antimicrobial resistance, selected virulence factors and genotypes of *S*. *aureus* from nasal carriage and clinical infection in Southwest Nigeria. Non-duplicate *S*. *aureus* isolates were obtained from infection (n = 217) and asymptomatic carriers (n = 73) during a cross sectional study in Lagos and Ogun States, Nigeria from 2010–2011. Susceptibility testing was performed using Vitek automated systems. Selected virulence factors were detected by PCR. The population structure was assessed using *spa* typing. The *spa* clonal complexes (spa-CC) were deduced using the Based Upon Repeat Pattern algorithm (BURP). Resistance was higher for aminoglycosides in clinical isolates while resistances to quinolones and tetracycline were more prevalent in carrier isolates. The Panton-Valentine leukocidin (PVL) was more frequently detected in isolates from infection compared to carriage (80.2 vs 53.4%; p<0.001, chi^2^-test). Seven methicillin resistant *S*. *aureus* isolates were associated with *spa* types t002, t008, t064, t194, t8439, t8440 and t8441. The predominant *spa* types among the methicillin-susceptible *S*. *aureus* isolates were t084 (65.5%), t2304 (4.4%) and t8435 (4.1%). spa-CC 084 was predominant among isolates from infection (80.3%, n = 167) and was significantly associated with PVL (OR = 7.1, 95%CI: 3.9–13.2, p<0.001, chi^2-^ test). In conclusion, PVL positive isolates were more frequently detected among isolates from infection compared to carriage and are associated with spa-CC 084.

## Introduction

*Staphylococcus aureus* is a versatile human pathogen causing infections ranging from relatively mild skin and soft tissue infections to life threatening sepsis, pneumonia, osteomyelitis, endocarditis as well as toxin mediated diseases such as toxic shock syndrome and food poisoning. It is usually a colonizer of about one third of healthy humans and is most likely found in the nares, particularly in the posterior vestibules [1]. Nasal carriage of *S*. *aureus* is the source and a risk factor for staphylococcal disease [2,3]. Treatment of *S*. *aureus* is aggravated by the global spread of methicillin-resistant *S*. *aureus* (MRSA) [4].

Several studies have characterized *S*. *aureus* isolates from Nigeria but very few have compared the virulence genes and genotypes of isolates from infection and carriage in one study. Such a comparison is important as there is evidence, that some virulence factors and genotypes are more prevalent in African isolates from infection compared to colonization [5,6]. In particular, this holds true for the Panton-Valentine leukocidin (PVL) which is highly prevalent in *S*. *aureus* from infection but is less frequently found in isolates from colonization in Africa [7]. PVL can be associated with skin and soft tissue infection but its role in the pathogenesis of disease remains controversial [8,9]

Therefore, the objective of this study was to compare the antibiotic susceptibility, virulence factors and genotypes of community acquired *S*. *aureus* from infection and colonization from two states (Lagos and Ogun) in Southwest Nigeria in a cross sectional study.

## Materials and Methods

### Ethical Statement

A documented oral informed consent was obtained from all participants in this study. This was because a written informed consent would have been the only documentation linking the participants to the study as participants were willing to participate on conditions of anonymity. The consent was documented after the participants had understood the research and expressed willingness to participate. The consent procedure as well as this study was specifically approved by the ethics committee of the Department of Biological Sciences, Covenant University, Ota, Nigeria (CUNG-2010–035).

### Bacterial Isolates

Between 2010 and 2011, non-duplicate *S*. *aureus* isolates were obtained from eight medical centres; in Lagos State (Lagos University Teaching Hospital, Akoka (11.5%, n = 25), Lagos State University Teaching Hospital, Ikeja (9.7%, n = 21), General Hospital, Ikorodu (21.7%, n = 47), General Hospital, Ijede (24.4%, n = 53), Federal Neuro-Psychiatric Hospital, Yaba (5.1%, n = 11)) and in Ogun State (Covenant University Health Centre, Ota (5.5%, n = 12), Medicare Hospital Ota (8.3%, n = 18), Sacred Heart Hospital, Abeokuta (13.8%, n = 30)). Isolates were obtained from skin and soft tissue infections, urinary tract infection, respiratory tract infection, wounds, vaginitis, otitis media, conjunctivitis and septicaemia. Patients were excluded if positive cultures were obtained 48 hours after admission. Nasal swabs were obtained from the anterior nares of 120 asymptomatic hospital personnel (doctors, nurses and nursing assistants) and other asymptomatic carriers (University students) using sterile cotton swabs. The samples were cultured on mannitol salt and Columbia blood agar and *S*. *aureus* was presumptively identified using Gram staining, catalase production and slide coagulase test. Further confirmation was carried out with the latex agglutination test (Pastorex Staph-Plus, Bio-Rad Laboratories, Marnes-la-Coquette, France) according to the manufacturer’s instructions. The VITEK 2 system was used to confirm *S*. *aureus* isolates with the aid of the gram-positive (GP) identification card (bioMérieux, Marcy l'Etoile, France). In addition, the *S*. *aureus* specific thermostable nuclease (*nuc*) was detected by PCR for species confirmation [10].

### Antimicrobial susceptibility testing

Antimicrobial susceptibility testing was performed using Vitek-2 automated systems (bioMérieux). Minimum inhibitory concentrations were interpreted applying EUCAST clinical breakpoints (www.eucast.org). The antibiotics tested included benzylpenicillin, oxacillin, gentamicin, tobramycin, levofloxacin, moxifloxacin, erythromycin, clindamycin, linezolid, teicoplanin, vancomycin, tetracycline, tigecycline, fosfomycin, nitrofurantoin, fusidic acid, mupirocin, rifampicin and trimethoprim-sulfamethoxazole.

The resistance to penicillin and methicillin was confirmed by the PCR detection of *blaZ* and *mecA*, respectively [11,12]. The staphylococcal cassette chromosome *mec* (SCC*mec*) was typed using a multiplex PCR approach as described [13].

### Virulence factors

Capsular polysaccharides (*cap 5*, *cap 8*), exfoliative toxins (*eta*, *etb)*, the toxic shock syndrome toxin *(tst)* and the *luk*S-PV/*luk*F-PV genes, encoding the Panton-Valentine leucocidin (PVL) were detected by multiplex PCR [14–16].

The expression of most *S*. *aureus* virulence factors is controlled by the accessory gene regulator (*agr)* locus, which is characterized by a polymorphism of its autoinducing peptide that divides *S*. *aureus* isolates into four major groups. The *agr* types of the *S*. *aureus* isolates were determined by a multiplex PCR strategy [17].

### Genotyping

All isolates were *spa* typed based on the highly polymorphic region X of the protein A gene, which is composed of a variable number of 24-bp repeats [18]. The *spa* types were determined with the StaphType software version 2.2.1 (Ridom GmbH, Münster, Germany). *spa*-clonal complexes (spa-CC) were assigned using the based upon repeat pattern (BURP) algorithm as implemented in StaphType using preset parameters as recommended ("exclude *spa* types that are shorter than 5 repeats" and "*spa* types are clustered if costs are less or equal than 4") [18]. This algorithm clusters related *spa* types in spa-CC based on similarities of the repeat patterns in the polymorphic region X of the protein A gene. The spa-CCs are congruent with multilocus sequence typing (MLST) [18].

### Statistical analysis

Categorical variables (e.g. virulence factors, genotypes) were compared between isolates from infection and colonization using the chi^2^ test or Fisher’s exact test where appropriate. The association between categorical variables, the corresponding odds ratio (OR), 95% confidence interval (95%CI) and the significance level (p<0.05) were calculated with “R” (version 2.13.1) and the package “epicalc”.

## Results

### *S*. *aureus* isolates

A total of 290 *S*. *aureus* isolates were obtained from asymptomatic carriers (n = 73) and clinical infections (n = 217). Isolates were recovered from skin and soft tissue infections (22.6%, n = 49) urinary tract infections (20.7%, n = 45), respiratory tract infections (9.7%, n = 21), wound infections (22.1%, n = 48), vaginitis (0.9%, n = 2), otitis media (21.1%, n = 46), conjunctivitis (1.8%, n = 4), septicaemia (0.9%, n = 2).

### Antimicrobial resistance

All isolates possessed the *S*. *aureus* specific *nuc* gene. Resistance was highest for penicillin confirmed by the presence of *blaZ* (97.2%, n = 211 of clinical isolates and 97.3%, n = 73 of carrier isolates) followed by trimethoprim/sulfamethoxazole (84.3%, n = 183 of clinical isolates and 68.5%, n = 50 of carrier isolates); and tetracycline (13.8%, n = 29 of clinical isolates and 29.3% n = 22 of carrier isolates, Table 1). Of all the *S*. *aureus* isolates, 2.4% (n = 7) exhibited methicillin resistance. No resistance was detected against erythromycin, clindamycin, linezolid, glycopeptides, fusidic acid, mupirocin and rifampicin. All MRSA isolates carried the *mecA* gene and the SCC*mec* typing identified one MRSA isolate to contain SCC*mec* type I (t8439, PVL positive) while another contained SCC*mec* type IV (t8440, PVL positive). The SCC*mec* was not typeable in all other MRSA (n = 5).

**Table 1:** Comparison of *Staphylococcus aureus* from infection and colonization in Nigeria, 2010–2011.

	Antibiotics	Clinical isolates (n = 217), n (%)	Carrier isolates (n = 73), n (%)	Total (n = 290), n (%)	OR, 95% CI	p-value
**Antimicrobial resistance**	Penicillin	211 (97.2)	73 (100)	284 (97.9)	1.0 (0.2–10.5)	1.0
	Oxacillin	5 (2.3)	2 (2.7)	7 (2.4)	1.2 (0.1–7.5)	1.0
	Gentamicin	10 (4.6)	1 (1.4)	11 (3.8)	0.3 (0.0–2.1)	0.301
	Tobramycin	10 (4.6)	1 (1.4)	11 (3.8)	0.3 (0.0–2.1)	0.301
	Levofloxacin	12 (5.5)	11 (15.1)	23 (7.9)	3.0 (1.1–7.9)	0.021
	Moxifloxacin	10 (4.6)	11 (15.1)	21 (7.2)	3.7 (1.3–10.1)	0.003
	Tetracycline	29 (13.4)	22 (30.1)	51 (17.6)	2.8 (1.4–5.5)	0.001
	Tigecycline	1 (0.5)	0 (0)	1 (0.4)	0.0 (0.0–115.7)	1.0
	Fosfomycin	1 (0.5)	0 (0)	1 (0.4)	0.0 (0.0–115.7)	1.0
	Trimethoprim/Sulfamethoxazole	183 (84.3)	50 (68.5)	233 (80.3)	0.4 (0.2–0.8)	0.003
**Virulence factors**	PVL	174 (80.2)	39 (53.4)	213 (79.7)	0.3 (0.2–0.5)	<0.001
	*Eta*	0 (0)	2 (2.7)	2 (0.7)	0.0 (0.0–1.8)	0.063
	*Tst*	1 (0.5)	3 (4.1)	4 (1.4)	0.1 (0.0–1.4)	0.051
	*cap5*	13 (6.0)	7 (9.6)	20 (6.9)	0.6 (0.0.2–1.9)	0.294
	*cap8*	204 (94.0)	66 (90.4)	270 (93.1)	1.7 (0.5–4.7)	0.294
***agr* subtypes**	*agr* I	13 (6.0)	21 (28.8)	34 (11.7)	0.2 (0.1–0.4)	<0.001
	*agr* II	176 (81.1)	39 (53.4)	215 (74.1)	3.7 (2.0–6.9)	<0.001
	*agr* III	1 (0.5)	7 (9.6)	8 (2.8)	0.0 (0.0–0.4)	<0.001
	*agr* IV	27 (12.4)	6 (8.2)	33 (290)	1.6 (0.6–4.9)	0.326

### Virulence factors

The proportion of PVL-positive isolates was significantly higher in isolates from infection compared to colonization (80.2% (n = 174) vs. 53.4% (n = 39), OR = 0.3, 95%CI: 0.2–0.5, p<0.001). However, the presence of PVL was not associated with a certain entity of infection. For instance, no significant association was detected between PVL-positive isolates and skin and soft tissue infections, urinary tract infection, respiratory tract infection, wound infection and otitis media.

Of note, PVL possession was associated with resistance to trimethoprim/sulfamethoxazole (OR = 2.7, 95% CI: 1.4–5.3, p = 0.001) and penicillin (OR = 9.1, 95%CI: 1.5–100, p = 0.005).

The distribution of the remaining virulence factors was similar among isolates from infection and colonization (Table 1). The *etb* gene was not detected.

### Genotypes

All 290 *S*. *aureus* isolates fell into one of the four previously described *agr* groups (alleles). The subgroup *agr* II and *agr* IV were significantly more frequently detected among clinical isolates while *agr* I and *agr* III were more often found in isolates from colonization (Table 1). Thirty-seven *spa* types were identified in this study. The most prevalent was t084 (65.5%, n = 192), followed by t2304 (4.4%, n = 13) and then t8435 (4.1%, n = 12). Fourteen *spa* types were associated with the clinical isolates, the most prevalent was t084 (74.8%, n = 163), followed by t2304 (5%, n = 11) and then t8441 (4.6%, n = 10). *S*. *aureus* isolates from carriers belonged to 26 different *spa* types, the most prevalent was t084 (38.7%, n = 29), followed by t091 (10.7%, n = 8) and t1931 and t8435 (4%, n = 3, each) and t091 (10.7%, n = 8). Eleven novel *spa* types were identified from this study (t8435, t8436, t8437, t8438, t8439, t8440, t8441, t8442, t8952, t8953, t8954).

**Fig 1:**
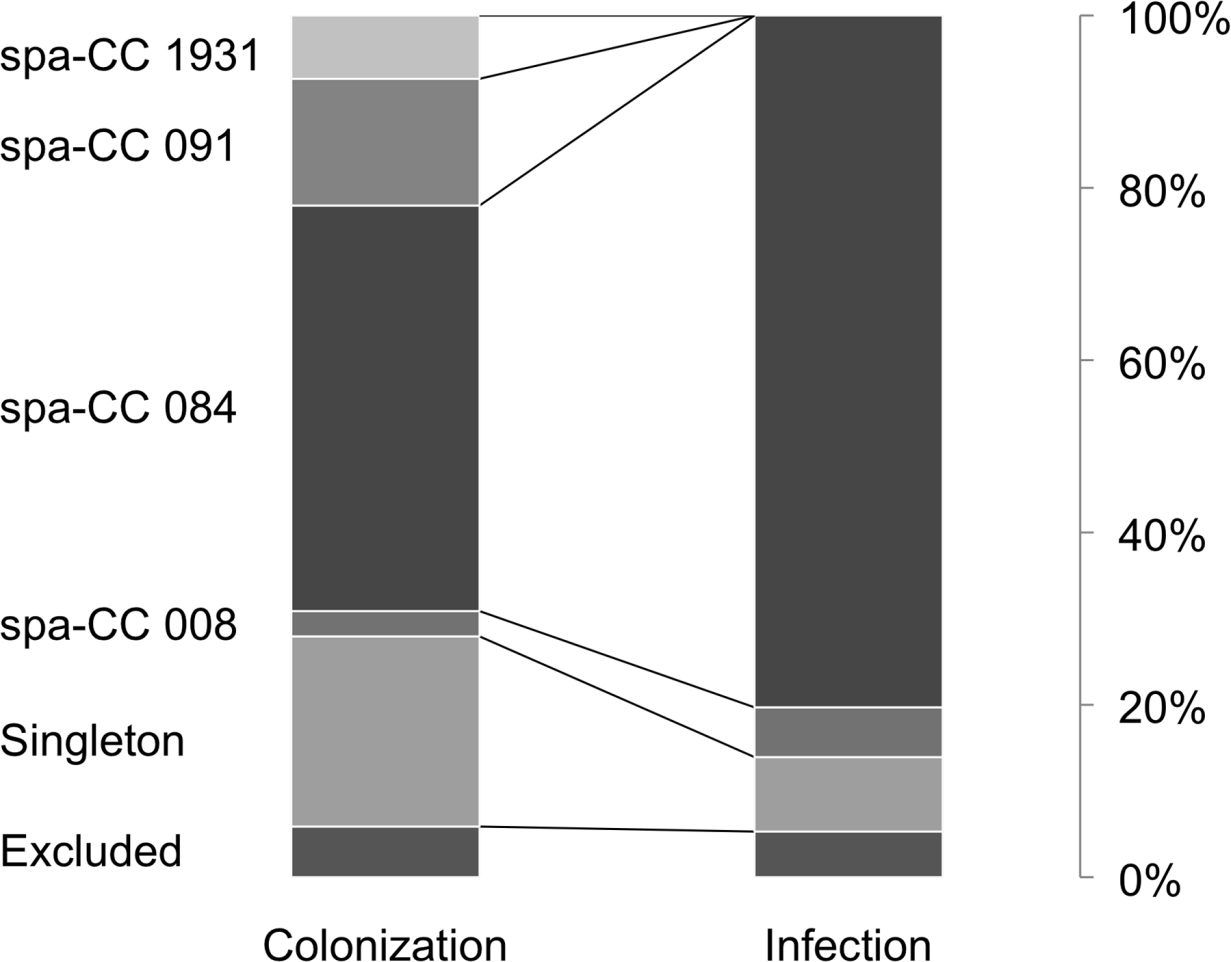
Population structure of Nigerian *Staphylococcus aureus* from colonization and infection. Stacked bars represent the proportion of spa-clonal complexes (spa-CC). Spa-CC 1931and spa-CC 091 are solely found in carrier isolates.

The BURP analysis showed an unequal distribution of spa-CC among insolates from colonization and infection. While spa-CC 1931 and spa-CC 091 were not detected in clinical isolates, spa-CC 084 was the most prevalent complex among isolates from infection accounting for 80.3% (n = 167, Fig 1). In our dataset, spa-CC 084 was significantly associated with PVL-positive isolates (OR = 7.1, 95%CI: 3.9–13.2, p<0.001). Noteworthy, all spa-CC 008 and spa-CC 091 isolates were PVL negative.

## Discussion

We report here a basic molecular characterization of *S*. *aureus* from colonization and infection in Nigeria. Main findings are a significant association of clinical isolates with PVL and spa-CC 084.

The proportion of isolates from urinary tract infection seems to be high (20.8%) in our study compared to Europe and Brazil (1.1%) [19]. In general, a high proportion of *S*. *aureus* from Africa is reported from urinary tract infections (6.3–13.9%), reasons for this phenomenon are unknown.

The proportion of MRSA among all *S*. *aureus* (2.4%) was slightly lower compared to other studies from Nigeria (11–11.5%) [20,21]. We detected two PVL positive MRSA (t8439, repeat pattern: 07–23–21–17–34–383–16–34–33–13 and t8440, repeat pattern: 07–13–12–12–34–34–33–212). We consider these MRSA as unrelated to the PVL-positive USA300 clone which is found in Africa and which belongs to t112 (repeat pattern: 11–19–19–21–17–34–24–34–22–25) and t121 (repeat pattern: 11–19–21–17–34–24–34–22–25) [5,22].

We report high rates of PVL-positive isolates which is in line with other studies from Africa. Sub-Saharan Africa is considered to be a PVL endemic region showing PVL prevalence among *S*. *aureus*, particularly methicillin susceptible isolates, of 17–74% [5]. Among other virulence factors (e.g. hemolysin, phenol soluble modulins), PVL can be associated with skin and soft tissue infections but its role in the pathogenesis of disease is still controversial [8,23,24]. Similar to a study from Gabon, we detected significantly more PVL-positive isolates among *S*. *aureus* from infection but did not find an association of PVL with skin and soft tissue infection [6].

In our dataset, PVL-positive isolates were also significantly more frequently resistant to trimethoprim/sulfamethoxazole and penicillin. A recent study from Gabon also showed a clear association of PVL with resistance to trimethoprim/sulfamethoxazole and it was suggested, that the use of antimicrobials including trimethoprim/sulfamethoxazole could select for PVL positive isolates [25]. Participants being colonized with PVL-positive isolates reported more frequently skin and soft tissue infections in this study from Gabon [25].

We found a clear dominance of spa-CC 084 among isolates from infection (Fig 1). This spa-CC usually belongs to the multilocus sequence type (ST) clonal complex CC15 commonly isolated in sub-Saharan Africa (e.g. Cameroon, Gabon, Madagascar, Nigeria, Niger, Senegal) [26–28]. Our finding is in contrast to a study from Gabon which did not show a significant association of ST15 with disease [6]. One reason for this conflicting results could be that we analysed more isolates from superficial infection (e.g. respiratory and urinary tract infection, vaginitis) while isolates from invasive infections (e.g. bloodstream infections, pyomyositis) were more dominant in the Gabonese study [6].

Although our analyses provide important information on the differences in antimicrobial resistance, selected virulence factors and genotypes between isolates from infection and colonization, few limitations need to be addressed. Firstly, our study might not be representative for the whole study region as we only included a small proportion of carrier isolates. In addition, clinical isolates were only collected at healthcare centres which may not be representative of the community. Secondly, some *S*. *aureus* were isolated from patients with vaginitis according to the physician’s judgment. However, it has to be emphasized that even though *S*. *aureus* can be found within the vaginal microbiota and it is usually not a typical pathogen causing vaginal infections (except e.g. from abscesses). Therefore, there could remain a risk that these isolates were rather colonizing than infecting isolates.

In conclusion, PVL positive isolates are more frequently detected among isolates from infection compared to carriage and are associated with spa-CC 084.
